# Catheter Ablation for the Management of Atrial Fibrillation: An Update of the Literature

**DOI:** 10.3390/life13081784

**Published:** 2023-08-21

**Authors:** Shahana Hussain, Catrin Sohrabi, Rui Providencia, Syed Ahsan, Nikolaos Papageorgiou

**Affiliations:** 1 Electrophysiology Department, Barts Heart Centre, St Bartholomew’s Hospital, London EC1A 7BE, UK; 2 Royal Free London NHS Foundation Trust, London NW3 2QG, UK; 3 Institute of Cardiovascular Science, University College London, London WC1E 6BT, UK

**Keywords:** catheter ablation, atrial fibrillation, heart failure

## Abstract

**Simple Summary:**

First-line treatment for restoring and maintaining sinus rhythm in patients with symptomatic atrial fibrillation (AF) involves the use of antiarrhythmic drugs. However, these are associated with significant side effects and have limited success rates in terminating AF episodes. In contrast, catheter ablation has been shown to be superior in maintaining sinus rhythm and improving the quality of life of AF patients. These procedures can also be performed via a hybrid set-up involving a minimally invasive surgical approach. These options have shown promise in restoring and maintaining sinus rhythm but are not without risks. Therefore, further studies investigating different ablative strategies are needed.

**Abstract:**

Catheter ablation has been shown to be more effective at maintaining sinus rhythm and improving quality of life when compared to antiarrhythmic drugs. Radiofrequency and cryoablation are two effective methods. However, catheter-only ablation strategies have not consistently produced high success rates in treating longstanding and persistent AF patients. The emerging treatment of choice for such cases is hybrid ablation, which involves a multidisciplinary and minimally invasive approach to achieve surgical ablation of the direct posterior left atrial wall in combination with endocardial catheter ablation. Studies have shown promising results for the hybrid approach when compared with catheter ablation alone, but it is not without risks. Large and randomised studies are necessary to further evaluate these strategies for managing AF.

## 1. Medical Management of Atrial Fibrillation

Antiarrhythmic drugs (AADs) are the first-line treatment of choice for restoring and maintaining sinus rhythm in patients with symptomatic atrial fibrillation (AF). In patients for whom rhythm control is not appropriate, rate control can be used instead by exerting a negative chronotropic effect to reduce the ventricular rate in combination with anticoagulation for stroke prevention. AADs have demonstrated debatable results with class 1 and 3A agents terminating 50% of AF episodes, whilst amiodarone is able to terminate only 70% of AF episodes [1]. They are also associated with significant side effects and can be proarrhythmic [2].

AF catheter ablation has become established as the superior alternative to maintaining sinus rhythm and achieving an improvement in quality of life. Catheter ablation also offers curative treatment in contrast to AADs, through electrical isolation of the pulmonary veins. Current guidelines recommend AF ablation in the event of AAD failure; however, the role of AF ablation as the first-line treatment option for AF is surfacing. At present, the European Society of Cardiology and American College of Cardiology guidelines provide a restricted recommendation for AF ablation as first-line therapy, limiting it to a highly selected patient group with symptomatic paroxysmal (class IIa) or persistent AF (class IIb) without major risk factors for AF recurrence such as left atrial size, AF duration, and renal dysfunction as well as consideration of patient preference [3,4]. Overall, these guidelines provide comprehensive information regarding indications to treatment strategies and relevant clinical data for patients with AF [3].

This review will examine current ablation management strategies in paroxysmal AF, heart failure in concomitant AF and persistent AF, and the evidence base behind these.

## 2. Radiofrequency Ablation as First-Line Treatment

The first catheter ablation in humans was performed in 1981 by Scheinman through the delivery of DC shocks to an electrode catheter [5]. This provided proof of principle for closed-chest catheter ablation for arrythmias; in particular, AF. Scheinman’s work was the foundation for the development of radiofrequency (RF) energy catheters allowing the delivery of precise lesions. Haissaguerre, a French electrophysiologist, later pioneered the use of RF catheter ablation in patients with AF by inserting the catheter into the pulmonary veins (PVs) of human hearts and mapping the triggers for AF [6]. He determined that the origin of these triggers was found in the PVs in 96% of patients. Through mapping and ablating these foci in the PVs, 62% of patients had freedom from AF without the need for AAD therapy. Haissaguerre also then developed a new strategy for PV isolation (PVI). In contrast to his earlier work of mapping individual triggers, he used a multielectrode circular catheter placed at the junction of the PV and the left atrium to localise these. He then electrically isolated the entire PV from the atrium, leading to the development of PV isolation as an empirical treatment for AF. This led to the current use of catheter ablation as a standard treatment for AF.

Three key trials have addressed the relative differences in ablation and pharmacological therapy as first-line interventions in paroxysmal AF (PAF). The Radiofrequency Ablation versus Antiarrhythmic Drugs as First-line Treatment of Symptomatic Atrial Fibrillation (RAAFT-1) trial was the first to suggest the merits of catheter ablation as a first-line therapy for AF. In this trial, 70 patients with symptomatic PAF (96% PAF; mean age 54 years) were randomised to either catheter ablation or AAD therapy (Table 1). The ablation protocol compromised of PV antrum isolation confirmed by recordings from a circular mapping catheter. In this early trial, only non-irrigated tip ablation catheters (8 mm) were used. At the end of 1-year follow-up, 63% of patients assigned to AAD therapy experienced ≥1 recurrence of symptomatic AF, as compared with 13% of those assigned to the PV antrum isolation arm. These figures accounted for an 80% relative risk reduction with catheter ablation (*p* < 0.001). In addition, PV antrum isolation was associated with a significantly lower rate of hospitalisation (9% versus 54%; *p* < 0.001) and improved quality of life.

It is crucial to highlight that the benefits of catheter ablation as shown in the study may have been underestimated, given the high rate of crossover to catheter ablation in patients initially assigned to AAD (51%). Complications were also comparable between the two treatment groups (12.5% in the catheter ablation arm versus 11.5% in the AAD group).

Given the results of the RAAFT trial, two further large multicentre randomised trials assessed radiofrequency ablation (RFA) as first-line therapy over AAD: the Medical Antiarrhythmic Treatment or Radiofrequency Ablation in Paroxysmal Atrial Fibrillation (MANTRA-PAF) trial and the Radiofrequency Ablation versus Antiarrhythmic Drugs as First-Line Treatment of Paroxysmal Atrial Fibrillation (RAAFT-2) trial.

MANTRA-PAF randomised 294 patients with symptomatic PAF to either RFA or AADs. In contrast with the RAAFT trial, the ablation methods used were heterogeneous and varied from PV isolation guided by circular mapping catheter to circumferential PV ablation guided by a three-dimensional electroanatomic mapping system (CARTO, Biosense-Webster, Diamond Bar, CA, USA). The utilisation of different ablation methods was at the discretion of the enrolling cardiologist. The primary study endpoint was cumulative AF burden (symptomatic and asymptomatic) during 7-day Holter recordings after 3, 6, 12, 18, and 24 months of follow-up. Freedom from any AF after 24 months, quality of life, and burden of symptomatic AF were included among the secondary endpoints. The trial failed to meet its primary endpoint of a reduction in the cumulative burden of AF >2 years; however, it still demonstrated that CA was associated with a lower rate of AF recurrence compared with AAD (15% versus 29%, respectively; *p* = 0.004).

In contrast with RAAFT, the treatment failure of MANTRA-PAF in achieving its primary endpoint may be due to its discretional use of circumferential ablation without confirmation of PV isolation with a circular mapping catheter. This strategy has shown inferiority to PV isolation confirmed by a circular mapping catheter [7]. It is also important to note that 36% of patients allocated to AAD crossed over to the RFA arm, as outcomes were analysed according to the intention-to-treat principle, and this may have further impacted the results.

The RAAFT-2 trial utilised similar inclusion criteria, endpoints, and ablation techniques as RAAFT; however, it had a significant exception with the utilisation of irrigated-tip ablation catheters. A total of 127 patients with symptomatic PAF were randomised to either RFA or AAD. After 2 years, AF/atrial tachycardia recurred in 54.5% of the RFA group compared with 72.1% of the AAD group (*p* = 0.016) at follow-up. The RAAFT-2 trial also had a substantial number of patients crossing over from AAD to RFA (26%).

These three studies used distinct ablation techniques; however, MANTRA-PAF and RAAFT-2 demonstrated an overall low level of success rates for freedom from atrial arrhythmias in the ablation arm. There was also no procedural standardisation between studies, and endpoints differed. AAD use also differed post-ablation in all studies: in RAAFT-1, beta blocker therapy was administered post-ablation as per physician preference. In RAAFT-2, AADs were only administered during the 90-day blanking period post-ablation if required, and MANTRA-PAF allowed AADs in the initial 3 months post-ablation. Furthermore, the outcomes of point-by-point PVI by RF ablation are dependent on operator proficiency given the complexity of creating contiguous curvilinear ablation lesions using catheters initially developed for ablating focal arrhythmic targets [8].

## 3. Cryoablation as First-Line Treatment

Another approach to isolate PVs in AF involves the use of a cryoballoon. In contrast to PVI procedures, cryoballoon ablation is considered faster to perform compared to PVI since the point-by-point creation of individual lesions around each pulmonary vein may take longer to successfully achieve. Moreover, cryoballoon ablation is generally regarded as easier to learn and perform for electrophysiologists, as opposed to precise catheter manipulation and careful lesion creation in PVI. However, there is concern regarding higher radiation exposure during fluoroscopy. The introduction of cryoballoon technology in 1999 was a breakthrough invention as operators were able to achieve electrical isolation of the PVs with one single application, in contrast to the point-by-point lesion set delivered by RFA, thereby making it more appealing. The Arctic Front Balloon (Medtronic CryoCath) was the first cryoballoon technology used for ablation. This involved the use of the double-walled 10.5Fr cryoballoon catheter, which would inflate and fill with coolant (nitrous oxide) to deliver ablation lesions between the PVs and the LA. The 15Fr FlexCath Steerable Sheath was a deflectable delivery sheath that would allow introduction of the cryoballoon into the LA. The CryoConsole was the third component of the cryoballoon technology, which contained the coolant and mechanical components. The nitrous oxide would then be delivered to the double-walled balloon where it would undergo a liquid-to-gas transformation, delivering cooling temperatures of approximately −80 °C.

There were three major randomised trials comparing cryoballoon ablation to AADs as first-line therapy of AF. These include the Catheter Cryoablation Versus Antiarrhythmic Drug as First-Line Therapy of Paroxysmal Atrial Fibrillation (Cryo-FIRST) trial [9], the Early Aggressive Invasive Intervention for Atrial Fibrillation (EARLY-AF) trial [10], and the Cryoballoon Catheter Ablation in an Antiarrhythmic Drug Naive Paroxysmal Atrial Fibrillation (STOP-AF First) trial [11] (Table 1).

These three studies included a total of 724 patients in their intention-to-treat. Across them, the mean ages were 60 years (STOP-AF First), 60 years (EARLY-AF), and 52 years (CRYO-First). Most patients were male (61%, STOP-AF First; 68%, CRYO-First; 69%, EARLY-AF) and the majority of patients had normal left ventricular function and left atrial size. Although the patients had been enrolled at an early stage of diagnosis (median time from first AF diagnosis of 12 months), patients were mostly high symptomatic from their arrhythmia on the basis of their Atrial Fibrillation Effect on QualiTy-of-life (AFEQT) scores (mean score 60.1). Three-minute freezes were standardised in EARLY-AF, while they were recommended in STOP-AF First and Cryo-FIRST. Procedure duration was shortest in Cryo-FIRST (84 ± 29 min), moderate in EARLY-AF (106 min (IQR: 89–131 min)), and longest in STOP-AF First (139 ± 74 min; *p* < 0.0001). Class Ic AADs were the initial AAD of choice used in the antiarrhythmic drug group (first agent prescribed in 92% in Cryo-FIRST, 82% in EARLY-AF, and 79% in STOP-AF First). In EARLY-AF, 30% of the AAD group needed several antiarrhythmic drug trials to achieve suppression of AF on implantable monitoring. Subtherapeutic antiarrhythmic drug dosing was observed in 7% in Cryo-FIRST, 0% in EARLY-AF, and 21% in STOP-AF First, with 18%, 0%, and 12% permanently discontinuing the study drug, respectively.

There was no crossover from AADs to ablation before the occurrence of a primary endpoint event in EARLY-AF, but it occurred in Cryo-FIRST (14%) and STOP-AF First (15%). STOP-AF and CRYO-FIRST assessed tachyarrhythmia occurrence with 12-lead electrocardiography at scheduled time points and 24 h Holter monitoring during months 6 to 12. Meanwhile, EARLY-AF monitored atrial tachyarrhythmias with continuous cardiac rhythm monitoring with an implantable cardiac device.

In contrast to the results of previous studies of first-line RFA, first-line cryoablation consistently demonstrated significant reductions in arrythmia recurrence (Table 1). EARLY-AF demonstrated that after one year, atrial tachyarrhythmia recurrence occurred in 42.9% of the patients who underwent ablation versus 67.8% of the patients receiving drug therapy (HR 0.48, 95% CI 0.35–0.66, *p* < 0.001). Cryoballoon ablation was also associated with lower recurrence of symptomatic atrial tachyarrhythmia (11.0% versus 26.2%) and a significant reduction in AF burden. The rate of serious adverse events was similar in the two groups (3.2% versus 4.0%). STOP-AF showed that initial success of the ablation procedure was achieved in 97% of patients, and procedure-related serious adverse events were uncommon. The percentage of patients with treatment success at 12 months (freedom from initial failure of the procedure or atrial arrhythmia recurrence after day 90) was higher with ablation than with drug therapy (74.6% versus 45.0%; *p* < 0.001). CRYO-FIRST showed freedom from atrial arrhythmias was higher in patients who underwent cryoablation compared to the AAD treatment arm (82.2% of versus 67.6%, HR = 0.48, *p* = 0.01). The incidence rate of symptomatic palpitations was notably reduced in the cryoballoon compared to the AAD arm (7.61 days/year versus 18.96 days/year; IRR = 0.40, *p* < 0.001).

It is important to note that each study employed different rhythm monitoring protocols, which could impact clinical outcomes. Intermittent non-invasive cardiac rhythm monitoring was used in Cryo-FIRST and STOP-AF, whereas EARLY-AF used implantable cardiac monitoring. Non-invasive monitoring is less sensitive in the detection of paroxysmal arrhythmias and can exaggerate the approximations of arrhythmia-free survival. EARLY-AF was the only study to provide AF burden data as it employed implantable cardiac monitoring, where it demonstrated a significant reduction in AF burden with ablation. However, the cardiac monitor was implanted when treatment was initiated so it did not evaluate change in AF burden from baseline. Additionally, continuous monitoring captured a higher AF recurrence.

These substantial randomised trials offer definitive evidence that AF ablation is superior to AADs as first-line treatment of patients with paroxysmal AF. The outcomes from the pivotal STOP-AF trial also formed the basis for the U.S Food and Drug Administration (FDA) approval for the Arctic Front CryoAblation Catheter system for the treatment of drug refractory PAF in 2010.

## 4. Radiofrequency Ablation Versus Cryoablation as First-Line Treatment

Two randomised studies directly compared both cryoablation and RFA for the treatment of drug-refractory PAF. These include FIRE AND ICE and CIRCA-DOSE. Both showed that these two approaches are comparable in terms of efficacy and safety.

In the FIRE AND ICE trial, a total of 762 patients were randomised to each arm with an average follow-up of 18 months (Table 2). The study did not find any significant difference between the two methods with regards to the efficacy endpoint. Additionally, both were comparable in terms of safety outcomes. A subsequent modified intention-to-treat analysis showed that cryoballoon was superior to RFA in the rate of repeat ablations, cardioversions, and cardiovascular re-hospitalisation during follow-up. Moreover, it demonstrated that in patients who underwent re-ablation, the number of electrical conduction relapses between the pulmonary veins and the left atrium was substantially reduced when the cryoballoon was used for the initial PV isolation at enrolment. This may be explained by cryoballoon catheters having a higher stability compared to RF catheters. Moreover, it is important to highlight that the adoption of new catheter technology was not uniform in the study. Specifically, the second-generation cryoballoon was used in 75.6% of patients in the cryoballoon arm, while only 24.7% of patients in the radiofrequency catheter group received advanced-generation catheters. This imbalanced use of second-generation cryoballoon technology versus advanced-generation catheters could have masked the true efficacy of the advanced contact-force sensing technology. Although the primary efficacy outcome did not show a difference between catheter generations, it remains essential to discern the impact of recently introduced technology on overall clinical outcomes, given the influx of new technologies in the field of AF ablation.

The cryoballoon was also shown to be more cost-effective due to lower resource utility post-ablation and lower payer cost than the RF arm. In summary the FIRE AND ICE trial confirmed the non-inferiority of the cryoballoon to RF ablation in patients with PAF. It also demonstrated a similar safety profile. Prior to this study, the European Society of Cardiology and the Heart Rhythm Society recommended radiofrequency energy as the leading ablation energy source. Following the trial, both societies updated their consensus documents to advocate for both cryoablation and RFA as a safe and efficient modality for the treatment of AF.

The CIRCA-DOSE also directly compared both ablation modalities and its findings were in support of the FIRE AND ICE trial. However, there were two main differences. First, both RF and cryoablation were performed with the latest-generation catheter (contact force catheter and cryoballoon). CIRCA-DOSE further compared two cryoablation regimens (4 min versus 2 min freezes) to the use of contact-force-guided RF to isolate the PVs in patients with PAF. Secondly, all patients underwent implantation of a loop recorder. There were no significant differences between the two different ablation approaches in terms of recurrent atrial arrhythmias’ occurrence in the first year. However, it did show an overall >98% reduction in arrhythmic load, but with no significant difference between the different ablation methods in the intention-to-treat analysis.

Furthermore, no significant difference in 1-year efficacy was seen between the 2 min and the 4 min freeze procedures; however, procedure duration was significantly reduced with the 2 min freeze protocol. Overall, the CIRCA-DOSE trial demonstrated that PVI completed either by cryoballoon or contact-force-guided RFA resulted in comparable freedom from atrial arrhythmias, as observed by implantable cardiac monitoring.

## 5. AF and Heart Failure

AF and heart failure often concomitantly occur in the same patients and have a complex interlinked relationship, in which the presence of one can cause and compound the other. The chronology carries significance for the prognosis and management of the patient. A meta-analysis of 53,969 patients established that in patients with heart failure, AF was associated with a 14% risk of death in patients included in observational studies (*p* < 0.05) and a 40% increased risk of death in patients included in randomised clinical trials (*p* < 0.0001) [12].

A tachycardia-induced cardiomyopathy can result from the consequences of the atrial tachycardia through complex electrophysiological, structural, neurohormonal and contractile changes within the atria. During AF, the absence of atrial systole can decrease left ventricular filling and decrease cardiac output by as much as 25% [13]. In addition, the tachycardia and loss of a coordinated circulation due to the irregularity can increase the probability of developing a cardiomyopathy [14]. This is usually a reversible form, as has been observed in patients who develop left ventricular dysfunction during AF; after restoration of sinus rhythm, the LV function recovers. The pro-BNP levels have also been shown to reduce dramatically in these patients, which can help distinguish from other forms of cardiomyopathies such as dilated cardiomyopathy [15]. Heart failure is also associated with an increased likelihood to develop atrial fibrillation and progress to persistent AF.

## 6. AF Treatment Strategies in the Context of Heart Failure

AAD therapy is often used to maintain sinus rhythm and prevent recurrence of AF. Currently amiodarone is the only guideline-recommended antiarrhythmic agent in heart failure [3], but it is associated with many long-term risks and drug interactions. The AF-CHF trial (Atrial Fibrillation and Congestive Heart Failure) was the largest trial to study rate versus rhythm control; it randomised 1376 patients with LVEF < 35% to either rate control or pharmacological rhythm control [16]. Patients in the rhythm control arm underwent electrical cardioversion if they did not revert to sinus rhythm after 6 weeks’ use of AADs (amiodarone, sotalol, and dofetilide). Patients randomised to the rate control arm were treated with beta blockers and digoxin; if rate control was still poor, then atrioventricular nodal ablation and pacemaker therapy was recommended. The study found no significant benefit of rhythm over rate control as there were no significant differences in all-cause mortality or secondary outcomes in either arm. Thus, the study outlined the difficulty in maintaining sinus rhythm and limited clinical benefit in AAD use in heart failure patients.

AAD use is also challenging because of its negative inotropic effects and multiple poorly tolerated side effects. As such, significant data have shown catheter ablation as the preferred treatment approach in patients with AF and heart failure.

## 7. PVI and Heart Failure

Many trials have been undertaken to assess the efficacy of catheter ablation in heart failure. The Pulmonary Vein Antrum Isolation versus AV Node Ablation with Bi-Ventricular Pacing for Treatment of Atrial Fibrillation in Patients with Congestive Heart Failure (PABA-CHF) trial was the first randomised study to directly compare AVN ablation with biventricular pacing to PVI ablation in patients with drug-resistant AF and heart failure with reduced ejection fraction (HFrEF) (Table 3) [17]. It showed that patients who underwent PVI had a significantly higher probability of being free of AF at 6 months, and they were more likely to achieve improvement in multiple functional parameters of heart failure such as quality-of-life scores, ejection fractions, and 6 min walk scores (*p* < 0.05).

MacDonald et al. randomised 41 patients with severe LVSD and persistent AF to rate control or PVI catheter ablation, and they measured changes in the LVEF as the primary endpoint [18]. In the rate control group, if the heart rate was above 80 beats/min, digoxin was added. The study did not show any significant improvement in LVEF or the 6 min walk test in the PVI group; this may be due to a higher proportion of patients with advanced heart failure (90% were NYHA III or IV).

The ARC-HF (catheter ablation versus rate control in the management of persistent atrial fibrillation in heart failure) trial [19] and CAMTAF (catheter ablation versus medical treatment of atrial fibrillation in heart failure) [20] trial compared PVI catheter ablation with pharmacological rate control. Both studies had limited follow-up: 12 months and 6 months. Both trials also had relatively small patient numbers (52 and 50 patients, respectively). The CAMTAF trial also showed that there was a substantial improvement in LV function and functional capacity in those patients that underwent PVI after 6 months compared to rate control. The ARC-HF trial assessed peak oxygen consumption as its primary endpoint; it demonstrated that PVI was associated with a significant increase in peak oxygen consumption at 12 months (*p* = 0.018).

The CAMERA-MRI study (catheter ablation versus medical rate control in atrial fibrillation and systolic dysfunction) included 66 patients with persistent AF and idiopathic cardiomyopathy with an EF < 45% [21]. After enrolment, patient rate control was optimised and they then underwent cardiac MRI imaging (CMR) to assess the LVEF and ventricular fibrosis using late gadolinium enhancement. Patients were then randomised to either treatment arm, and then they underwent a repeat CMR after 6 months. The primary endpoint was change in LVEF on repeat CMR imaging. The study found that patients randomised to catheter ablation had improvement in their LVEF (18 ± 13%) compared to the medical rate control group (4.4 ± 13%) (*p* < 0.0001). The catheter ablation treatment arm also noted a reduction in fibrosis on CMR at 6 months in those who were restored to sinus rhythm.

There are now data available on the long-term outcomes of this trial with 4 years of follow-up [22]. At 6 months following completion of the trial, patients in the rate control group were offered the opportunity to undergo catheter ablation; 18 patients from the original 33 allocated to rate control crossed over to catheter ablation. They noted an absolute increase in the LVEF in those that underwent catheter ablation by 16.4 + 13% compared to 8.6 + 7.6% in the rate control group (*p* = 0.001). The study noted, at 4.0 ± 0.9 years of follow-up in the catheter ablation group, the absence of ventricular LGE on CMR correlated with LVEF normalisation in 19 patients (58%) versus 4 patients (18%; *p* = 0.008) who exhibited late gadolinium enhancement on their CMR.

The Ablation Versus Amiodarone for Treatment of Persistent Atrial Fibrillation in Patients With Congestive Heart Failure and an Implanted Device (AATAC) trial randomised 603 patients (Table 3). The trial concluded CA was associated with a higher freedom of AF (70% versus 34%, *p* < 0.001) after a follow-up of 24 months [23]. Patients also had a lower mortality (31% versus 57%, *p* < 0.001) and a reduction in unplanned hospitalisation (8% versus 18%; *p* = 0.037) in the PVI ablation group.

Catheter ablation in heart failure patients gained popularity after the landmark CASTLE-AF (Catheter Ablation vs. Standard Conventional Therapy in Patients with Left Ventricular Dysfunction and Atrial Fibrillation) trial. It randomised 363 patients with symptomatic AF, HF and a LVEF < 35%, New York Heart Association class II–IV, and an implantable defibrillator to either PVI or medical therapy (rate or rhythm control). Median LVEF was 25% with a median follow-up of 37.8 months. The trial demonstrated that patients randomised to catheter ablation had a reduction in the composite endpoint of all-cause mortality or HF hospitalisation (28.5% versus 44.6%, *p* = 0.007). This reduction in the composite endpoint was independent of the degree of LV systolic impairment. Furthermore, patients with the least severe functional status demonstrated the greatest improvement. AF burden was considerably reduced in patients undergoing PVI. In addition, an AF burden of below 50% correlated with a lower incidence of the primary endpoint (*p* = 0.014).

The CABANA (Catheter Ablation versus Antiarrhythmic Drug Therapy for Atrial Fibrillation) trial randomised patients to either catheter ablation or antiarrhythmic drugs (AADs) [24]. The trial concluded that catheter ablation was neutral when compared to AADs in the primary composite endpoint of death, disabling stroke, serious bleeding, or cardiac arrest. The HF post hoc analysis has been recently published, which shows an improvement in outcomes in the PVI treatment arm (Table 3). It is important to note that of the 778 patients enrolled in the CABANA trial with heart failure, only 9.3% had an LV ejection fraction <40% and only 35% had New York Heart Association class > II at baseline. Therefore, it is important to interpret the results cautiously.

In 2020, the EAST-AFNET 4 trial (Early Treatment of Atrial Fibrillation for Stroke Prevention Trial) demonstrated that an early rhythm control strategy was superior to usual care in improving cardiovascular outcomes at 5 years in patients with early-onset AF (within 12 months) [2] The trial enrolled 2789 patients at 135 centres, with the choice of rhythm control at the discretion of the clinical centre. The rhythm control arm used flecainide as its initial rhythm control strategy in 36% of patients followed by amiodarone (20%) and catheter ablation (8%) at enrolment. The trial was stopped early due to efficacy.

The prespecified subgroup analysis has now become available in patients enrolled with heart failure in the EAST-AFNET 4 trial [25]. The study enrolled 798 patients with heart failure, with the majority having preserved LVEF (442 patients) and 132 patients with reduced ejection fraction LVEF <40%. The primary outcome was a composite of death from cardiovascular causes, stroke, hospitalisation with worsening HF or acute coronary syndrome. Patients with heart failure randomised to early rhythm control had a lower risk of the primary outcome (23.7%) compared patients randomised to usual care (32.3%); however, the difference was not significant (*p* = 0.03). It is important to note that most patients in the heart failure cohort had preserved LV function (56%). The study did find, however, that the most benefit found in terms of improvement in NYHA class was in those patients with HFpEF.

The results of these trials indicate that a reduction in the AF burden and restoration of sinus rhythm can result in improvements in prognosis, quality of life and left ventricular function in a select group of patients with heart failure and AF. PVI was the cornerstone of AF ablation in all these trials, and no randomised trial has investigated the use of different ablation strategies in patients with heart failure.

## 8. Catheter Ablation as First-Line Treatment in Persistent AF

In PAF, most of the foci appear to be the PVs and, therefore, PVI has shown great success in reducing arrhythmia recurrence, where the FIRE AND ICE trial resulted in greater than 75% freedom from atrial arrhythmias at 12 months in both the cryoballoon and RFA arms. The CIRCA-DOSE trial reported an approximate 50% freedom from atrial arrhythmias at 12 months, but with the use of an implantable loop recorder.

In patients with PAF with recurrence, this is commonly due to recovered pulmonary vein conduction requiring further PVI ablation. Patients with longstanding persistent AF are conversely very difficult to maintain in sinus rhythm with success rates of 40% to 75% at 12 months, with further decline over time [26,27,28].

To date, there have been no randomised control studies assessing first-line catheter ablation in persistent AF patients published. The upcoming RAAFT-3 trial (Radiofrequency Ablation versus Antiarrhythmic Drugs for Atrial Fibrillation Treatment) will be crucial in providing guidance as to whether first-line RFA can be applied to persistent AF patients.

## 9. Ablation Strategies in Persistent AF

The underlying principle of AF ablation is to achieve complete pulmonary vein isolation, either by cryoablation or RFA. Achievement of permanent PV isolation is challenging, with PV reconnection rates reported as high as 70% [26,27].

Although there are no specific consensus recommendations in ablation strategy in persistent AF patients other than complete PVI, the European consensus documents advocate for more extensive ablation such as isolation of the LAA, SVC isolation, linear lesions in the atria, ganglionic plexus lesions, fibrosis-guided voltage and so on in persistent and longstanding persistent AF patients [3].

The BELIEF (Left Atrial Appendage Isolation in Patients With Longstanding Persis-tent AF Undergoing Catheter Ablation) trial was a randomised study assessing the bene-fit of LAA isolation for the treatment of longstanding persistent AF [29]. Patients were randomised to an extended PV antrum ablation plus non-PV trigger ablation versus ex-tended PV antrum ablation plus non-PV trigger ablation plus empirical LAA isolation. The trial concluded empirical isolation of the LAA along with extended ablation reduced recurrence of arrhythmia at 12 months (56% versus 28%, *p* = 0.001). Overall, there were improved outcomes with targeting extrapulmonary foci, in particular, the LAA as adjunctive to PVI, without increasing complication risk.

Another randomised single-centre study by Kircher et al. demonstrated individually tailored substrate modification guided by voltage mapping significantly improved arrhythmia-free survival rates in ablation-naive patients in both the paroxysmal and persistent AF populations [30].

Linear ablation is the creation of contiguous lesions representing lines of electrical block to compartmentalise the LA, to reduce the ability of the cardiomyocytes to conduct electrical circuits responsible for AF propagation and maintenance. Successful linear ablation is confirmed by bidirectional block across the ablation lines. These lines include the LA roofline, the posterior line and the mitral isthmus. Several studies have provided evidence for the benefits of linear ablation as an adjunct to PVI, especially in the persistent AF population [31,32,33]; however, other studies have shown disappointing long-term findings [34]. This can also be a challenging procedure requiring excellent technical operator skills; furthermore, incomplete block is common and is a principal cause of atrial tachycardias in the future.

The STAR AF II (Substrate and Trigger Ablation for Reduction of AF Trial Part II) trial was the largest randomised control trial to assess outcomes of different ablative strategies in persistent AF refractory to at least one AAD [31]. It compared three ablation strategies: PV isolation, PVI with ablation of complex fractionated electrograms, and PVI plus linear ablation. The freedoms from AF at 18 months were 59%, 49%, and 46%, respectively. Likewise, after two ablation procedures, there were no significant differences between ablation strategies and freedom from arrhythmia recurrence with or without AADs (PVI alone 72%, PVI + electrograms, 58% PVI + lines; *p* = 0.018). The trial concluded that performing additional, and possibly unnecessary, ablation lesions could increase the risk of complications. Additional lesion sets also elongated procedure time and fluoroscopy exposure.

More recently, the CAPLA (Catheter Ablation for Persistent Atrial Fibrillation: A Multicenter Randomised Trial of Pulmonary Vein Isolation Versus PVI with Posterior Left Atrial Wall Isolation) trial randomised 338 patients with persistent AF to either PVI with PWI or PVI alone. At 12 months of follow-up, the addition of PWI to PVI alone did not significantly improve freedom from atrial arrhythmias [35]. It should be noted that a more extended period of follow-up would have provided a more comprehensive assessment of the long-term advantages of PWI (or drawbacks of PVI alone). In the ERASE-AF (Low-Voltage Myocardium-Guided Ablation Trial of Persistent Atrial Fibrillation) trial, a total of 324 patients with persistent AF were randomised to either PVI alone or PVI plus substrate modification. At follow-up, PVI plus individualised ablation of atrial low-voltage myocardium was found to significantly improve outcomes in patients with persistent AF [36]. Collectively, these trials indicate that a personalised approach to persistent AF ablation may lead to improved results compared to a universal one-size-fits-all strategy.

## 10. Limitations of Current Ablation Methods in the Persistent AF Population

In patients with longstanding persistent AF, the triggers are from a diseased left atrial with multiple wavelets, macro re-entries and localised drivers. This requires substrate modification, defragmentation, and linear ablations to achieve optimal results.

To date, anatomical additive lesions such as targeting the mitral isthmus, left atrial roof, left atrial posterior wall, ganglionic plexus ablation or substrate-based ablation strategies such as targeting areas of low voltage or fibrosis have shown little added benefit [31,32,37]. Further work is being undertaken to assess the effects of left atrial appendage electrical isolation in reducing recurrence rates [33]. At present, no catheter-only-based ablation strategy has shown a consistent high rate of success in persistent AF patients.

The posterior wall of the left atrium shares a common embryological origin with the pulmonary veins. Therefore, emphasis has turned to targeting the posterior wall of the left atrium in extra-PV ablation. The posterior wall also carries the arrhythmogenic propensity for arrhythmias as the pulmonary veins and can, therefore, act as an anatomical substrate to initiate and potentiate AF. Isolation of the posterior wall of the left atrium with endocardial catheterisation has been attempted with varying strategies; however, there have been obstructions to successful posterior wall isolation using an exclusive endocardial catheter-based approach, such as the risk of damaging structures in close proximity to the posterior left atrium, including the oesophagus, phrenic lungs, and pulmonary structures.

## 11. Complications of Catheter Ablation

Prospective registry-based studies have shown 4–14% of patients undergoing catheter ablation to experience complications, of which 2–3% may be life threatening. These may include cardiac tamponade, oesophageal perforation, periprocedural death, or a periprocedural thrombo-embolic event. Other complications include those related vascular access [38]. When guided by vascular ultrasound, femoral vein puncture has been shown to decease the incidence of vascular complications, such as major bleeding, in patients undergoing ablation when compared to the traditional palpation-guided approach. This is widely regarded as a highly effective measure to enhance safety outcomes during ablation procedures [39,40].

The use of fluoroscopy is also associated with complications and prolonged exposure to ionising radiation poses a health risk to both the patient and medical staff. As such, the cumulative radiation exposure during multiple procedures or prolonged ablation sessions may increase the risk of tissue damage and long-term issues such as skin-related injuries and possible cancer [41]. Following the ALARA principle (as low as reasonably achievable) is, therefore, important to minimise radiation exposure [42].

Cryotherapy confers multiple advantages when compared to radiofrequency ablation. First, there is marginal disruption to the surface of the endocardium, producing lesions that are less thrombogenic. Second, conservation of the tissue integrity with cryoablation results in reduced complications such as oesophageal injury/fistula, cardiac perforation and tamponade, and pulmonary vein stenosis. Third, homogenous well-delineated lesions are produced, which are less arrhythmogenic than lesions produced with RFA.

Significant advances in imaging have made catheter ablation safer, such as the development of intracardiac echocardiography (ICE). During the ablation procedure, ICE images can be integrated with the electro-anatomical mapping system, which has shown reduction in radiation exposure through shortened fluoroscopy times and a reduction in overall procedural time [43]. It is also well tolerated by patients and does not require a second operator to be present during the procedure. ICE also enhances safety and efficacy of the procedure, providing real-time information to the operator to allow direct visualisation during the transeptal puncture, catheter tissue contact to optimise delivery of ablation lesions and catheter feedback to allow for catheter manipulation [44,45]. ICE is also able to detect the development of a pericardial effusion prior to changes in patient haemodynamics and can detect thrombus formation on catheters [45]. Progression in catheter-based technology has also improved the efficiency profile of catheter ablation, such as the use of high-power short-duration (HPSD) RFA [46]. Standard RFA strategies employ low to moderate power (25–35 watts) for long-duration settings (30–60 s per lesion). HPSD PVI was originally reported in 2006. This employs higher power (45–50 Watts) for shorter duration (2–10 s on the posterior wall, 5–15 s at other endocardial sites in the LA). Studies have shown HPSD RFA provided significantly shortened procedure duration, fluoroscopy exposure, and RFA time compared to conventional RFA with comparable safety outcomes [47,48]. In addition, the use of visualisable (via electroanatomical mapping systems) and steerable sheaths assists in mitigating radiation exposure [49], and zero-fluoroscopy catheter ablation procedures have been deemed feasible [50], including cases involving cryoballoon ablation [51].

## 12. Surgical AF Ablation

The initial cut-and-sew maze procedure was performed in 1987 [52]. Succeeding revisions, resulting in the Cox maze III and Cox maze IV, have yielded high success rates, maintaining sinus rhythm in 80–90% of patients off AADs [34]. However, the surgical maze procedure requires cardiopulmonary bypass and is associated with significantly higher morbidity compared with a catheter-based approach.

Therefore, the future of AF ablation has turned to a hybrid approach, turning to minimally invasive surgical ablation of the direct posterior left atrial wall in combination with endocardial ablation, as described in the following segment.

## 13. Hybrid Ablation: The Future of Persistent AF Treatment?

Hybrid ablation is emerging as the treatment of choice for longstanding AF through targeting the pulmonary veins and the posterior wall of the left atrium using a multidisciplinary approach. It is performed in two stages by electrophysiologists and surgeons through an integrated minimally invasive epicardial ablation followed by endocardial catheter ablation. The left atrial appendage also undergoes electrical isolation using a surgically applied clip or suture device.

The aim of the hybrid approach is to generate durable transmural lesions and close the left atrial appendage (LAA). This is then followed by endocardial catheter ablation, which validates the lesion sets and addresses the additional arrhythmogenic substrate to complete the PVI lesion set and any additional triggers. These procedures can be completed in a two-stage procedure, or even on the same day at centres with a dedicated hybrid theatre.

Currently, there are two ablation approaches: (a) the hybrid ablation approach and (b) the hybrid convergent ablation approach. The hybrid ablation approach uses thoracoscopic access and bipolar radiofrequency clamps followed by endocardial catheter ablation. The hybrid convergent procedure engages a subxiphoid incision to access the pericardial space. Using direct visualisation, an irrigated unipolar catheter is applied to the left atrial posterior wall to create parallel ablation lines across the posterior wall. This is then followed by the endocardial component.

### 13.1. Hybrid Ablation

Wolf et al. published the first outcomes of 21 patients who underwent the minimally invasive surgical approach using bilateral video-assisted thoracoscopic PVI with excision of the LAA, compared to standard catheter ablation [53]. All enrolled patients had failed AAD therapy or had intolerance to AAD or intolerance to vitamin K antagonist. This study paved the way for further studies and demonstrated the feasibility of a minimally invasive approach. As it showed, 91.3% of patients were free of atrial arrhythmias at 3 months; however, the follow up period was short. Many studies have since shown consistently improved outcomes in patients undergoing hybrid ablation as compared to endocardial catheter ablation, as summarised in Table 4; however, these studies have been comparative or observational, rather than randomised.

The outcomes from the first randomised clinical trial for hybrid ablation have recently been published. The Hybrid Ablation Versus Repeated Catheter Ablation in Persistent Atrial Fibrillation (HARTCAP-AF) trial randomised 41 patients with longstanding persistent AF to either hybrid ablation or catheter ablation [60]. They underwent PVI, posterior left atrial wall isolation and then, at the operator discretion, a cavotricuspid isthmus ablation. The primary outcome was freedom from any atrial tachyarrhythmia >5 min off antiarrhythmic drugs after 12 months. The study demonstrated that the hybrid ablation group had a significantly higher freedom from atrial arrhythmias when compared to the catheter ablation group (89% versus 41%, *p* = 0.002). They also showed no significant difference in adverse outcomes between each group. They reported one pericarditis requiring pericardiocentesis and one femoral arteriovenous fistula in the hybrid ablation group. In the catheter ablation arm, 1 bleeding from the femoral artery occurred. Although the trial was small, the results are encouraging in support of a hybrid approach for persistent AF patients.

### 13.2. Convergent Ablation

Kiser et al. performed the first hybrid convergent procedure in 2010. In contrast to traditional epicardial surgical ablation using bipolar clamps, a unipolar, irrigated RF catheter was used across a minimally invasive transdiaphragmatic approach utilising a pericardioscopic cannula with a guidewire [61]. Currently, the convergent ablation procedure is the least invasive hybrid AF ablation procedure available.

The initial epicardial lesion set Kiser et al. performed was extensive and resembled the extracardiac maze lesion set, which included linear ablation of the posterior PV antrum, anterior aspect of the PVs, along the coronary sinus, ligament of Marshall, and SVC. Following surgical closure, electrophysiological mapping was performed to guide endocardial catheter ablation, which was focused on PVI, coronary sinus isolation and cavotricuspid isthmus (CTI). The goal of the original combined procedure was to achieve isolation of all four pulmonary veins and posterior left atrium, ablate the coronary sinus and confirm block at the cavotricuspid isthmus. Patients had cardiac rhythm monitoring with 24 h Holter monitoring at 3 months, and 24 h or 7-day monitoring at 6 and 12 months. The study demonstrated 76% of patients were free of atrial arrhythmias and AADs. However, the limitations of intermittent cardiac rhythm monitoring as compared to implantable cardiac rhythm monitoring apply as mentioned in previous studies.

A further comparative study by Maclean et al. in 2020 assessed outcomes of 43 patients with longstanding persistent AF who underwent the convergent ablation procedure with 43 propensity-matched patients undergoing endocardial catheter ablation (Table 5) [62]. The study showed at 12 months, patients who underwent the convergent ablation had a significantly higher freedom from atrial arrhythmias compared to catheter ablation after a single procedure (60.5% versus 25.6%, *p* = 0.002). The outcomes were consistent even at 12 months off AADs; atrial arrhythmia freedom was higher in the convergent group (37.2% versus 13.9%, *p* = 0.025). Maclean et al. did, however, find a higher complication rate in the patients undergoing convergent ablation, which may be due to Maclean et al. using endocardial radiofrequency ablation whereas Kress et al. used cryoballoon in most of their cohort [63]. Kress et al. also performed the convergent ablation in one single procedure, whereas Maclean et al. performed a two-stage procedure. Overall, the findings are still in keeping with improved outcomes in the convergent ablation arms over endocardial catheter ablation.

In 2020, the landmark trial CONVERGE (Convergence of Epicardial and Endocardial Ablation for the Treatment of Symptomatic Persistent AF) was performed [64]. This was the first multicentre randomised controlled trial to compare the convergent ablation approach with endocardial catheter ablation in 153 persistent AF patients.

DeLurgio et al. introduced the contemporary epicardial lesion pattern, consisting of posterior wall homogenisation through the creation of parallel, overlapping rows of contiguous lesions using the most recent generation of unipolar RF catheter (EPi-Sense, AtriCure, Inc.) in a linear configuration. Similar to Maclean et al., the primary access technique also changed to a subxiphoid incision for cannula insertion. This avoided the potential for transdiaphragmatic hernias developing post-procedure.

Patients were randomised in 2:1 to hybrid convergent ablation versus endocardial catheter ablation, this was performed to obtain data for safety outcomes. In the catheter ablation group, PVI, roofline, and cavotricuspid isthmus ablation were performed. If AF persisted, then additional complex fractionated atrial electrogram ablation was performed as per physician discretion. At 12 months, the convergent ablation arm showed superiority with freedom from atrial arrhythmias on AADs as compared to catheter ablation alone (67.7% versus 50.0%; *p* = 0.036). These results were sustained off AADs, too (53.5% vs. 32.0%; *p* = 0.0128). Furthermore, at 18 months using 7-day Holter, 74% of patients in the hybrid convergent arm achieved over 90% AF burden reduction when compared to 55% with endocardial catheter ablation only (risk ratio, 1.34; *p* = 0.0395).

## 14. Conclusions

Atrial fibrillation is the most prevalent sustained tachyarrhythmia and is associated with a reduction in the quality of life and increased morbidity and mortality. Catheter ablation has become the curative treatment of choice for many patients over the last two decades, especially for paroxysmal AF and in those with heart failure with reduced ejection fraction significantly reducing AF burden and cardiovascular hospitalisations. In persistent and longstanding AF, successful outcomes are suboptimal as no single catheter ablation strategy has shown sustained prevention of tachyarrhythmia recurrence. Surgical AF ablations have shown potential in restoring and maintaining sinus rhythm in this subset of persistent AF patients; however, it is not feasible to perform this nationwide for persistent AF. The hybrid and convergent AF procedure has shown significant promise for treatment in this challenging cohort of patients through a minimally invasive approach. Given the magnitude of the AF burden worldwide, it is likely hybrid ablation will form a major part of cardiac surgeons’ and electrophysiologists’ clinical practice. Further randomised control trials are underway with adjunct ablative strategies such as left atrial appendage isolation, which show potential for success.

## 15. Future Directions

Catheter ablation has always been carried out with a thermal energy source: either cryotherapy or radiofrequency energy to deliver lesion sets. An exciting new development in pulsed field ablation, which uses a nonthermal energy source, has the potential to deliver highly selective lesions without injury to surrounding structures such as the oesophagus or phrenic nerve, as has been the main limitation with the traditional method of catheter ablation. The recent PULSED AF (Pulsed Field Ablation to Irreversibly Electroporate Tissue and Treat AF) multicentre study has shown PFA is effective in arrhythmia-free survival, with rates comparable to current ablation technologies with a low rate of primary safety adverse outcomes (0.7%) [65]. Further randomised studies with direct comparison to current catheter ablation strategies are needed to demonstrate efficacy and superiority.

## Figures and Tables

**Table 1:** Catheter ablation versus antiarrhythmic drugs in the treatment for PAF.

Study	Treatment Arms	Year	Patient Number	Median Follow-Up (Months)	AF Ablation Strategy	Paroxysmal-AF Cases	Definition of Recurrence	Freedom from AF Recurrence: CA	Freedom from AF Recurrence: AAD	*p*-Value
RAAFT-1	RFA CA as first line therapy	2005	70	12	PVI	96%	AF recurrence lasting > 15 s	87%	37%	*p* < 0.001
MANTRA-PAF	RFA CA as first line therapy in PAF	2012	294	24	PVI + additional lesions as per physician preference	100%	AF recurrence lasting > 1 min	85%	71%	*p* = 0.004
RAAFT-2	RFA CA as first line therapy in PAF	2014	127	24	PVI + additional lesions as per physician preference	98%	>30 s of AF/AT/AFL occurrence	53%	41%	*p* = 0.03
STOP-AF	Cryoballoon CA as second line therapy in PAF	2013	245	12	PVI	100%	>30 s of AF/AT/AFL occurrence	69.9%	7.3%	*p* < 0.001
EARLY-AF	Cryoballoon CA as first line therapy in PAF	2021	303	12	PVI	95%	>30 s of AF/AT/AFL occurrence	57.1%	32.2%	*p* < 0.001
Cryo-FIRST	Cryoballoon CA as first line therapy in PAF	2021	218	12	PVI + additional lesions (if incomplete PVI or focal trigger identification)	100%	>30 s of AF/AT/AFL occurrence	82.2%	67.6%	*p* = 0.01

AAD: antiarrhythmic drug; AF: atrial fibrillation; CA: catheter ablation; PAF: paroxysmal AF; PVI: pulmonary vein isolation; RFA: radiofrequency ablation.

**Table 2:** Cryoablation and RFA for the treatment of PAF.

Study	Treatment Arms	Year	Patient Number	Median Follow-Up (Months)	Definition of Recurrence	Freedom from Recurrence (12 Months)	Outcome	Monitoring Method	Complications
FIRE AND ICE	RFA vs. cryoballoon PAF	2016	762	18	>30 s, with 3-month blanking period	RFA 76.9% Cryoballoon: 78.7%	Cryoballoon is noninferior to RFA with similar safety profiles	Holter monitor	RFA 12.8% versus CBA 10.2% (*p* = 0.24)
CIRCA-DOSE	Contact force RFA vs. 4 min cryoballoon vs. 2 min cryoballoon in PAF	2019	346	12	>30 s, with 84-day blanking period	RFA 53.9% 4 min cryoballoon: 52.2% 2 min cryoballoon: 51.7%	No significant differences between ablation strategies in reducing recurrences	Implantable loop monitor	RFA 2.6% versus CBA (4 min) 5.2% versus CBA (2 min) 6%

CBA: cryoballoon ablation; PAF: paroxysmal AF; RFA: radiofrequency ablation.

**Table 3:** Catheter ablation studies in patient with heart failure.

Study	Treatment Arms	Year	Patient Number	Median Follow-Up (Months)	AF Ablation Strategy	Persistent AF Cases	LVEF (%)	Ischaemic HF	Outcome	Definition Recurrence
PABA-CHF	PVI vs. AVN ablation	2008	81	6	PVI ± linear lines	51%	27 ± 8	73%	PVI was superior (*p* < 0.001)	30 s of AF/AT
MacDonald	PVI vs. rate control (digoxin)	2011	41	6	PVI ± linear lines	100%	16 ± 7	50%	PVI did not improve LVEF	AF occurrence
ARC-HF	PVI vs. rate control	2013	52	12	Stepwise approach	100%	22 ± 8	33%	PVI was superior (*p* = 0.018)	AF occurrence
CAMTAF	PVI vs. rate control	2014	50	12	PVI ± linear lines ± CFAE	100%	32 ± 8	26%	PVI was superior (*p* = 0.015)	30 s of AF/AT
AATAC	PVI vs. amiodarone	2016	203	24	PVI ± linear lines ± CFAE	100%	29 ± 5	62%	PVI was superior (*p* < 0.0001)	AF occurrence
CAMERA-MRI	PVI vs. rate control	2017	66	6	PVI ± linear lines	72%	35 ± 10	0%	PVI was superior (*p* < 0.0001)	30 s of AF/AT
CASTLE-AF	PVI vs. medical therapy (rate or rhythm control)	2018	363	37	PVI ± linear lines	70%	33 (IQR 25–38)	46%	PVI was superior (*p* = 0.007)	30 s of AF/AT
EAST-AFNET 4	Early rhythm control vs. medical therapy (rate or rhythm control)	2020	2789	61	N/A	26%	N/A	N/A	Early rhythm control was superior (*p* = 0.005)	AF occurrence
CABANA-HF	PVI vs. medical therapy (rate or rhythm control)	2021	778	48	PVI ± linear lines	55%	55	22%	PVI was superior (*p* < 0.05)	AF occurrence

AF: atrial fibrillation; AVN: atrioventricular node; CFAE: complex fractionated atrial electrogram; HF: heart failure; LVEF: left ventricular ejection fraction; PVI: pulmonary vein isolation.

**Table 4:** Summary of clinical studies comparing the hybrid ablation procedure versus endocardial ablation.

Study	Year	Patient Number	Persistent AF Cases	Surgical Approach	Median Follow-Up (Months)	AF Ablation Strategy	Freedom from AF	Complications
Wolf et al. [53]	2005	27	33%	Bilateral thoracoscopic	6	PVI and LAAC	91.3%	1 major, 3 minor
Pison et al. [54]	2012	26	42%	Unilateral	12	PVI, CTI, SVCi, intercaval line, mitral line	83%	1 minor
La Meir et al. [55]	2013	63	0%	Unilateral	12	PVI, inferior line, roof line, isthmus, LAAC	91.4%	0
Pison et al. [56]	2014	78	63%	Unilateral	12	PVI, roof line, inferior line, mitral line, CTI, intercaval line, LAAC, GPa	82% persAF 76% PAF	6 minor
Bulava et al. [57]	2015	50	100%	Bilateral	12	PVI, roof line, inferior line, intercaval line, LAAC, GPa	94%	7 major, 10 minor
Richardson et al. [58]	2016	83	100%	Bilateral	12	PVI, roof line, inferior line, intercaval line, LAAC	71%	6 minor, 1 major
HISTORIC-AF	2017	100	100%	Fusion	12	Box lesion	88%	3 minor, 3 major
Maesen et al. [59]	2018	64	53%	Unilateral	36	PVI, roof line, inferior line, LAAC	80%	2 major, 1 minor
HARTCAP-AF	2020	41	100%	Unilateral	12	PVI, PWI + CTI	89%	2 major

AF: atrial fibrillation; CTI: cavotricuspid isthmus ablation; LAAC: left atrial appendage occlusion; PVI: pulmonary vein isolation; PWI: posterior wall isolation; GPa: ganglionic plexi ablation; SVCi: superior vena cava isolation.

**Table 5:** Convergent ablation clinical studies.

Study	Year	Patient Number	Persistent AF Cases	Surgical Approach	Median Follow-Up (Months)	AF Ablation Strategy	Freedom from AF in Convergent Group	Freedom from AF In Catheter Alone	*p*-Value	Complications
Kress et al. [63]	2017	133	100%	Transabdominal	16	PVI, CFAE, ± mitral line and roof line if AF persisted	72%	51%	0.01	2 major
Maclean et al. [62]	2020	43	100%	Subxiphoid	30.5	PVI ± linear lesion ± CFAE	60.5%	25.6%	0.02	5 major
CONVERGE	2020	102	100%	Subxiphoid	12	PVI, PWI, CTI	67.7%	50%	0.036	8 major

CTI: cavotricuspid isthmus ablation; CFAE: complex fractionated atrial electrograms; PVI: pulmonary vein isolation; PWI: posterior wall isolation.

## Data Availability

The data from this manuscript are derived from publicly available published clinical trial and study results.
